# Integrated Transcriptomic, Proteomic, and Network Pharmacology Analyses Unravel Key Therapeutic Mechanisms of Xuebijing Injection for Severe Acute Pancreatitis

**DOI:** 10.3390/ph18121866

**Published:** 2025-12-07

**Authors:** Linbo Yao, Xinmin Yang, Mei Yuan, Shiyu Liu, Qiqi Wang, Yongzi Wu, Wenjuan Luo, Xueying Wu, Wenhao Cai, Lan Li, Ziqi Lin, Juqin Yang, Tingting Liu, Robert Sutton, Peter Szatmary, Tao Jin, Qing Xia, Wei Huang

**Affiliations:** 1 West China Centre of Excellence for Pancreatitis, Institute of Integrated Traditional Chinese and Western Medicine, West China-Liverpool Biomedical Research Centre, West China Hospital, Sichuan University, Chengdu 610041, China; 2 West China Biobank, West China Hospital, Sichuan University, Chengdu 610041, China; 3 Liverpool Pancreatitis Research Group, Institute of Systems, Molecular and Integrative Biology, University of Liverpool and Liverpool University Hospitals NHS Foundation Trust, Liverpool L69 3GE, UK; 4 Department of Critical Care Medicine, West China Hospital, Sichuan University, Chengdu 610041, China; 5 Institutes for Systems Genetics & Immunology and Inflammation, Frontiers of Science Center for Disease-related Molecular Network, West China Hospital, Sichuan University, Chengdu 610200, China

**Keywords:** acute pancreatitis, Xuebijing injection, network pharmacology, transcriptomics, proteomics

## Abstract

**Background:** Xuebijing Injection (XBJ), a plant-derived traditional Chinese medicine administered as an injection, is widely used in clinical practice to treat various acute critical illnesses including severe acute pancreatitis (SAP). The mechanisms by which XBJ alleviates SAP remain elusive. **Methods:** Active components of XBJ were identified using UPLC-QTOF/MS. A mouse SAP model was established by intraperitoneal injections of cerulein (50 μg/kg/h × 7) followed by lipopolysaccharide (10 mg/kg). XBJ of 2.5, 5, and 10 mL/kg was co-administered twice after induction of SAP. The protective effects of XBJ on pancreatic acinar cells were further investigated in vitro. An integrated analysis of transcriptomic data from human and mouse blood, as well as mouse lung, combined with network pharmacology were employed to delineate the therapeutic mechanisms of XBJ on SAP, followed by pancreatic immunoblotting and proteomics validation. **Results:** Component analysis revealed 9 active ingredients of XBJ. XBJ at 10 mL/kg had the best effect and consistently decreased pancreatic, lung, and circulatory pro-inflammatory indices. XBJ dose-dependently reduced necrotic cell death activation. Transcriptomics, proteomics and network pharmacology analyses identified 14 key targets, with IL-17-related signaling pathways being the most significant. Experimental validation further confirmed that XBJ significantly reduced serum levels of key IL-17-related inflammatory cytokines (such as IL-17, IL-1β, IL-6, and TNF-α) and downregulated the mRNA expression of related inflammatory factors in pancreatic tissue. Virtual docking and surface plasmon resonance demonstrate that hydroxysafflor yellow A had the highest binding affinity with MMP-9, MAPK14, and LCN2. Crucially, subsequent pancreatic immunoblotting and proteomics analyses did not confirm significant direct modulation of these targets at the protein level within pancreatic tissue. **Conclusions:** XBJ attenuates SAP severity by quelling pro-inflammatory mediators, an effect chiefly attributed to modulating systemic IL-17–related signaling rather than direct pancreatic intervention.

## 1. Introduction

Acute pancreatitis is a frequent and potentially life-threatening inflammatory disorder of the pancreas that affects millions of individuals worldwide each year with increasing incidence [1]. The severity and outcomes of acute pancreatitis are affected by the extent of pancreatic damage and organ failure due to persistent systemic inflammatory response syndrome. About 80% of acute pancreatitis patients experience mild disease which resolves in under a week of supportive treatment. Most mortality and morbidity occur in the remaining 20% patients who follow a severe clinical course complicated by organ failure and pancreatic necrosis [2]. When multiple and persistent organ failure occurs, especially in association with infected pancreatic necrosis or sepsis, a high mortality of over 30% ensues [3]. Ongoing phase II randomized controlled trials (RCTs) of pharmacological therapies targeting calcium release-activated calcium channels and tumor necrosis factor-alpha (TNF-α) aim to prevent further pancreatic necrosis and/or respiratory failure [4]. Nevertheless, new pharmacological therapies capable of reducing the impact of established organ failure are still lacking.

Xuebijing Injection (XBJ) is a Chinese herbal medicine (CHM) preparation composed of five herbal extracts: *Carthamus tinctorius* flowers (Honghua), *Paeonia lactiflora* roots (Chishao), *Ligusticum chuanxiong* rhizomes (Chuanxiong), *Angelica sinensis* roots (Danggui), and *Salvia miltiorrhiza* roots (Danshen) [5]. XBJ was approved for the treatment of sepsis and organ failure by the China Food and Drug Administration in 2004 followed by ratification for market approval as a drug product. A recent systematic review [6] evaluated the efficacy and safety of RCTs of XBJ for the treatment of patients with acute pancreatitis including those with severe acute pancreatitis (SAP). The team concludes XBJ reduced the duration of severity of symptoms and abnormal laboratory parameters compared to control and particularly reduces abdominal pain and distension. While these findings are yet to be validated in internationally registered and well-designed RCTs, it is plausible to elucidate precise mechanisms underlying the therapeutic value of XBJ on SAP in parallel. We hypothesize that the therapeutic efficacy of XBJ in SAP is primarily mediated through systemic immune and inflammatory modulation, a mechanism consistent with its multi-component nature and established clinical use in managing systemic inflammatory conditions.

In this study, proof-of-principle in vivo and in vitro experiments were conducted to investigate the therapeutic value of XBJ on experimental SAP in mice. The combination of transcriptomics, proteomics and network pharmacology analyses were also employed to further screen, validate, and characterize key therapeutic targets and pathways of XBJ in SAP. We propose this approach is of value for the research of CHM formulas and complex diseases, not only guiding future drug development for SAP but offering valuable insights applicable to similar studies in other diseases.

## 2. Results

### 2.1. Identification of Active Ingredients and Targets Prediction of XBJ

**Figure 1:**
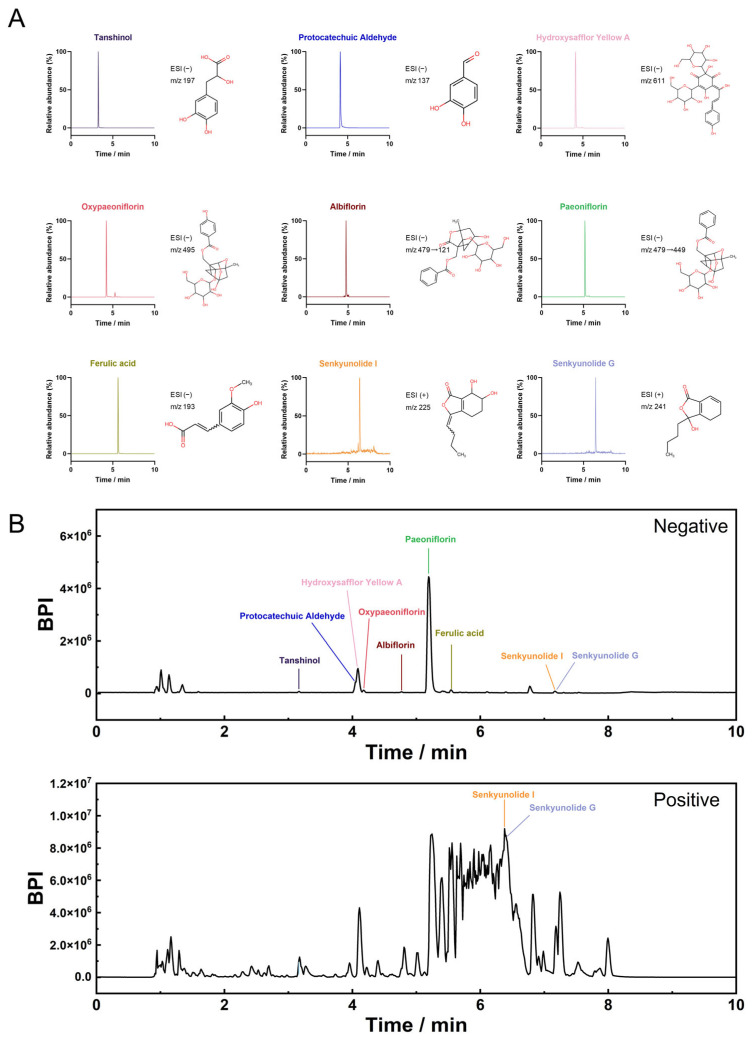
Total ion chromatogram fingerprint. (**A**) Standard components. (**B**) XBJ in negative and positive ion mode.

Representative chromatograms and chemical structures of standards for identified compounds are shown in Figure 1A. Total ion chromatogram fingerprint obtained by UPLC-QTOF/MS with both positive and negative ion modes are shown in Figure 1B. The nine compounds HSYA, paeoniflorin, albiflorin, oxypaeoniflorin, senkyunolide I, senkyunolide G, ferulic acid, tanshinol, and protocatechuic aldehyde were classified as representative active ingredients of XBJ. Nine representative active ingredients of XBJ were identified, encompassing diverse chemical classes: the complex C-glycosyl quinochalcone hydroxysafflor yellow A, the terpene glycosides paeoniflorin, the monoterpene glycosides albiflorin and oxypaeoniflorin, the natural phthalides senkyunolide I and senkyunolide G, the hydroxycinnamic acid ferulic acid, the polyphenolic acid tanshinol, and the phenolic aldehyde protocatechuic aldehyde. Using virtual screening, the known chemical properties of XBJ constituents were analyzed with several tools, including SwissTargetPrediction, TCMSP, ETCM, HERB, and TM-MC (Figure S1A). These platforms identified potential targets based on the structural similarity between XBJ constituent compounds and known bioactive molecules, resulting in 503 candidate target proteins. Subsequent GO (Figure S1B) and KEGG (Figure S1C) enrichment analysis of these target proteins revealed that they were primarily involved in pathways related to responses to stimuli and inflammatory responses. Specifically, this candidate target set included key inflammatory and proteolytic regulators such as TNF-α, IL-6, IL-1β, and MMP9, providing strong initial evidence for XBJ’s anti-inflammatory potential in SAP. These findings suggest that the therapeutic effects of XBJ on SAP may be mediated through the modulation of these key biological processes.

### 2.2. XBJ Attenuates Pancreatic and Lung Injury in SAP Mice

**Figure 2:**
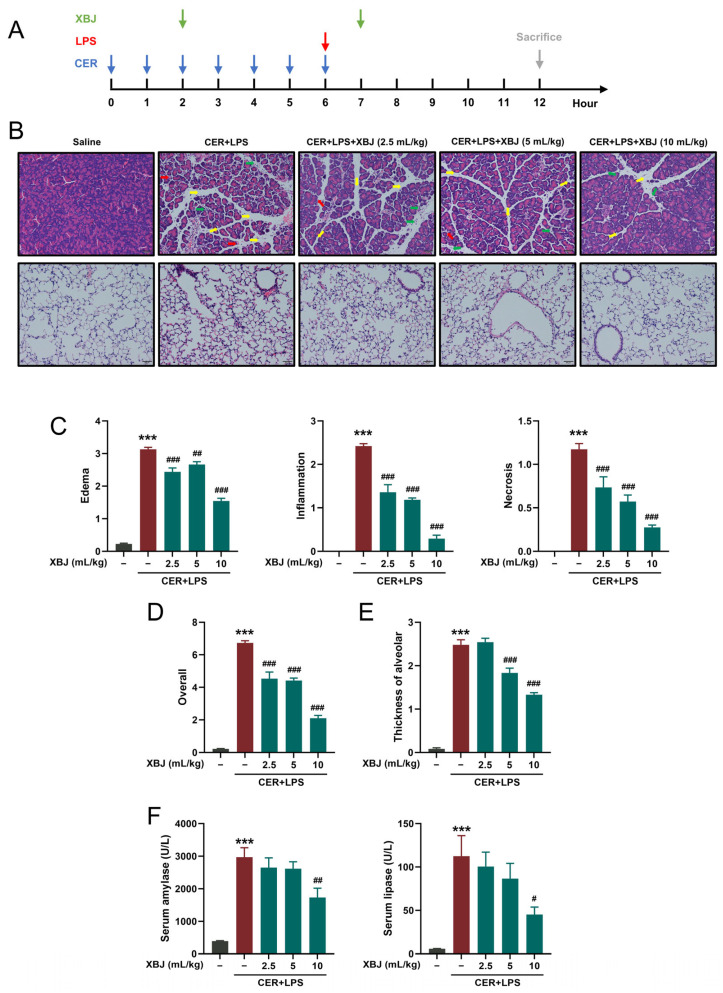
XBJ attenuates pancreatic and lung injury in SAP mice. (**A**) Experimental scheme for establishment of SAP model and administration of XBJ. (**B**) Representative H&E images of pancreatic and lung sections (scale bar = 50 μm, magnification ×200). Yellow arrows indicate edema, green arrows indicate inflammation, and red arrows indicate acinar cell necrosis. (**C**) Pancreatic histopathology scores (edema, inflammatory infiltration, and parenchymal necrosis) and (**D**) The overall score. (**E**) Alveolar wall thickness scoring in lung tissue. (**F**) Levels of serum amylase and lipase. All data are presented as means ± SEM of 6 mice per each experimental group. *** *p* < 0.001 indicates SAP vs. Control; ^#^ *p* < 0.05, ^##^ *p* < 0.01, and ^###^ *p* < 0.001 indicate SAP + XBJ vs. SAP.

The experimental procedure of the SAP model and XBJ administration regimen is shown in Figure 2A. The pancreas histopathology changes in SAP manifested as diffuse edema, marked periductal and parenchymal neutrophil filtration indicating inflammation, and scattered PAC necrosis with increased corresponding histopathological scores (Figure 2B–D) and concomitantly increased alveolar septal thickening score (Figure 2E). Compared with the control group, SAP had dramatically elevated serum amylase and lipase levels (Figure 2F). XBJ dose-dependently reduced biochemical indices and histopathological scores with only 10 mL/kg significantly and consistently alleviated severity of SAP (Figure 2B–F). Thus, the 10 mL/kg dose was selected as the most effective therapeutic dose for subsequent experiments, a selection based primarily on favorable histopathological and biochemical outcomes observed in the dose–response study. This concentration also aligns with XBJ dosages that have shown superior therapeutic effects in the treatment of other systemic inflammatory conditions [7].

### 2.3. XBJ Reduces Inflammatory Responses in SAP Mice

**Figure 3:**
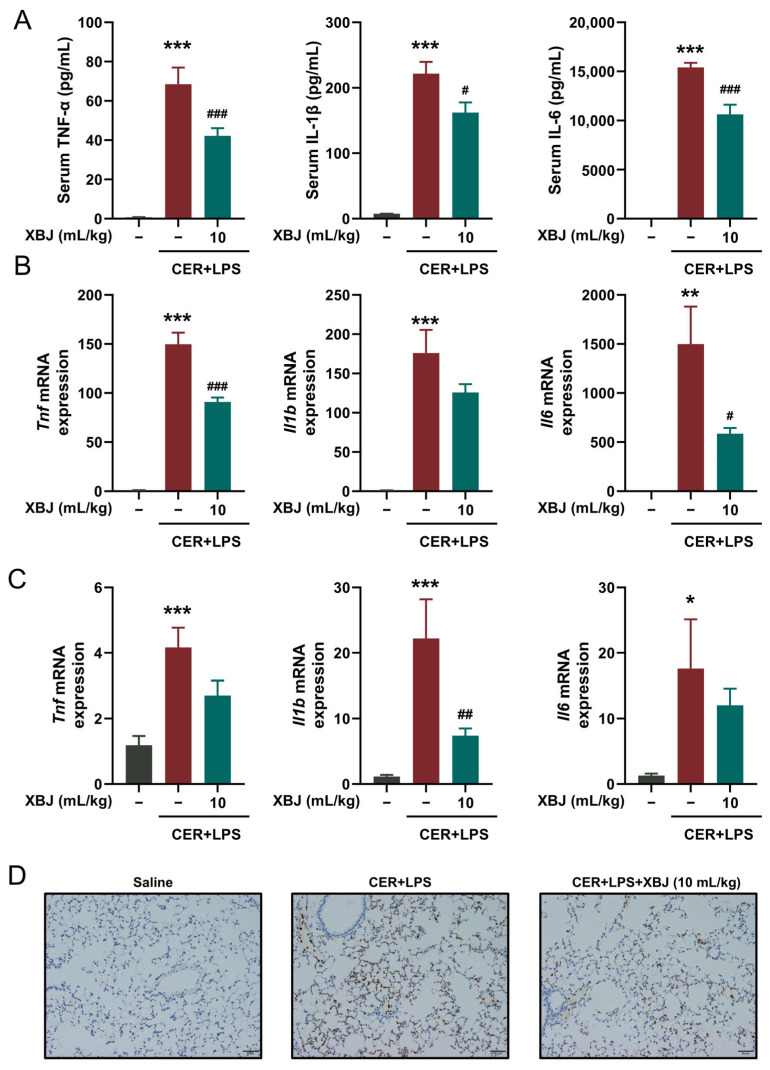
XBJ reduces systemic inflammation in SAP mice. (**A**) Levels of serum proinflammatory cytokines (TNF-α, IL-1β, and IL-6; *n* = 6 per group). (**B**) Expression of proinflammatory marker mRNAs (*Tnf*, *Il1b*, and *Il6*) in the pancreatic tissue (*n* = 3–4 per group). (**C**) Expression of proinflammatory marker mRNAs (*Tnf*, *Il1b*, and *Il6*) in the lung tissue (*n* = 3–4 per group). (**D**) Representative immunohistochemical staining for myeloperoxidase in the lung tissue (scale bar = 50 μm, magnification ×200). All data are presented as means ± SEM. * *p* < 0.05, ** *p* < 0.01, and *** *p* < 0.001 indicate SAP vs. Control; ^#^ *p* < 0.05, ^##^ *p* < 0.01, and ^###^ *p* < 0.001 indicate SAP + XBJ vs. SAP.

To evaluate the anti-inflammatory effects of XBJ in SAP, its therapeutic efficacy was assessed by measuring key inflammatory markers in both serum and tissue samples. First, serum levels of pro-inflammatory cytokines, including TNF-α, IL-1β, and IL-6 were significantly reduced in the XBJ-treated group compared to the SAP model group (Figure 3A), suggesting that XBJ alleviates systemic inflammation. In addition, mRNA expression levels of *Tnf*, *Il1b*, and *Il6* were markedly downregulated in both pancreatic (Figure 3B) and lung (Figure 3C) tissues of XBJ-treated mice, further indicating its anti-inflammatory effects at the tissue level. Furthermore, immunohistochemistry analysis of lung tissues showed a significant reduction in myeloperoxidase staining in the XBJ treated group (Figure 3D), indicating decreased neutrophil infiltration and inflammation in the lungs. These results collectively demonstrate that XBJ effectively attenuated inflammation in both the pancreata and lungs in the SAP model, highlighting its potential as an anti-inflammatory therapeutic agent for SAP.

### 2.4. XBJ Alleviates PAC Injury

**Figure 4:**
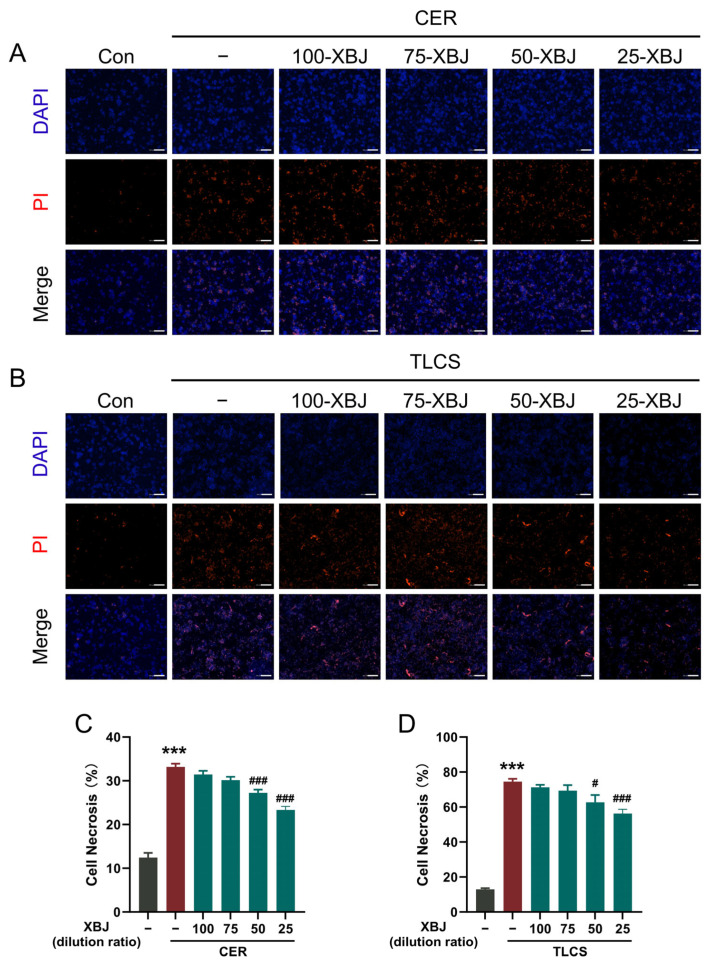
XBJ alleviates pancreatitis toxin-induced PAC injury. (**A**) CER- and (**B**) TLCS-induced necrotic cell death activation in PACs that were with or without concomitant treatment by various concentrations of XBJ. Representative images of PACs with DAPI (blue) and PI (red) staining (scale bar = 200 μm). Quantification of necrotic cell death (%) induced by (**C**) CER and (**D**) TLCS. 100-XBJ, 75-XBJ, etc., indicate 1:100, 1:75 (*v*/*v*), and other serial dilutions of the XBJ used in this experiment. All data are presented as means ± SEM of 4 individual samples per each experimental group. *** *p* < 0.001 indicates SAP vs. Control; ^#^ *p* < 0.05 and ^###^ *p* < 0.001 indicate SAP + XBJ vs. SAP.

The protective effects of XBJ on necrotic cell death activation of freshly isolated mouse PACs were investigated using two representative pancreatitis toxins—cholecystokinin analog cerulein (Figure 4A) and bile acid TLCS (Figure 4B). Cell necrosis was assessed using DAPI and PI staining, with varying concentrations of XBJ applied for intervention. The results show a significant increase in PI staining in cells treated with cerulein or TLCS, reflecting an increase in cell death. However, when treated with different concentrations of XBJ, lower doses exhibited minimal protective effects, with only a slight reduction in PI and DAPI staining observed. In contrast, higher concentrations of XBJ led to a marked reduction in PI uptake, suggesting a significant decrease in cell death. Statistical analysis revealed that the baseline PI uptake in control cells was approximately 13%. After cerulein or TLCS treatment, the PI uptake significantly increased to around 33% (Figure 4C) and 75% (Figure 4D), respectively. However, following XBJ treatment, PI uptake decreased, and the protective effect became more pronounced with increasing XBJ dosage. These findings demonstrate that XBJ effectively mitigated cerulein- and TLCS-induced PAC damage in a dose-dependent manner, with higher concentrations offering stronger protective effects.

### 2.5. Network Pharmacology Analysis of XBJ in SAP Based on Human Blood Transcriptomics

Collectively, our data provides strong evidence that XBJ exerts a protective effect in an experimental SAP model. However, the underlying molecular mechanisms by which XBJ acts remain unclear. To understand how XBJ exerts its protective effects in AP, we first analyzed the blood transcriptome of SAP patients to identify DEGs linked to potential drug targets of XBJ. As shown in Figure S2A, the distinct clustering of healthy controls and SAP group indicates significant differences in their overall phenotype. A total of 2691 DEGs were identified (Figure S2B). The results in Figure S2C demonstrate that the expression levels of the most significantly upregulated and downregulated genes can effectively distinguish SAP patients from healthy controls, suggesting their potential as biomarkers for accurate SAP diagnosis. GO (Figure S2D) and KEGG (Figure S2E) enrichment analyses further revealed that these genes are primarily involved in protein processing and infection-related pathways, highlighting their roles in protein homeostasis and immune regulation.

**Figure 5:**
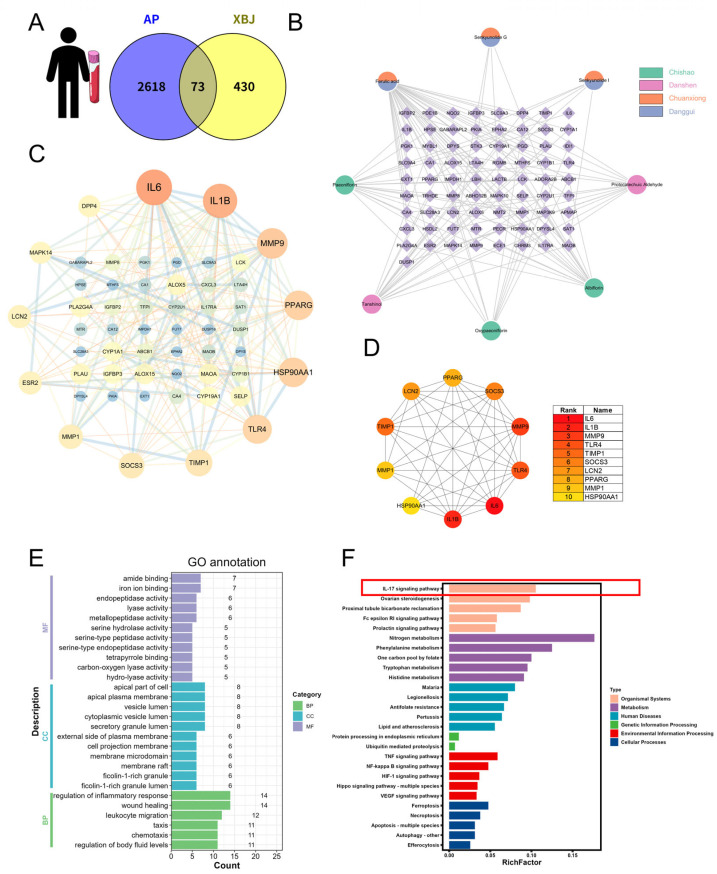
Network pharmacology analysis of XBJ in SAP based on human peripheral blood transcriptomics. (**A**) Intersection of “XBJ compound targets” and “SAP targets” in the human blood. (**B**) The compound–target network in the human blood. (**C**) PPI network. (**D**) Targets of the hub gene in human blood during SAP. (**E**) GO functional annotation. (**F**) KEGG pathway enrichment analysis.

Subsequently, network pharmacology was employed to predict the potential SAP targets of XBJ, integrating human blood transcriptome data. Using specific screening criteria, we identified 73 common proteins shared by both XBJ targets and SAP-related DEGs (Figure 5A). The corresponding compounds and herbs for these 73 target proteins are presented in Figure 5B. A PPI analysis of these 73 proteins was performed and visualized as a network (Figure 5C). The MCC algorithm from the CytoHubba plug-in was used to identify 10 candidate hub genes from the PPI network, including *IL-6*, *IL-1B*, *MMP9*, *TLR4*, *TIMP1*, *SOCS3*, *LCN2*, *PPARG*, *MMP1*, and *HSP90AA1* (Figure 5D). GO and KEGG enrichment analyses revealed that many target genes were strongly associated with the “regulation of inflammatory response,” “leukocyte migration,” “IL-17 signaling pathway,” “TNF signaling pathway,” and “NF-κB signaling pathway” (Figure 5E,F). These pathways are closely linked to inflammation, providing a solid foundation for further exploration of the underlying molecular mechanisms.

### 2.6. Network Pharmacology Analysis of XBJ in SAP Based on Mouse Blood Transcriptomics

**Figure 6:**
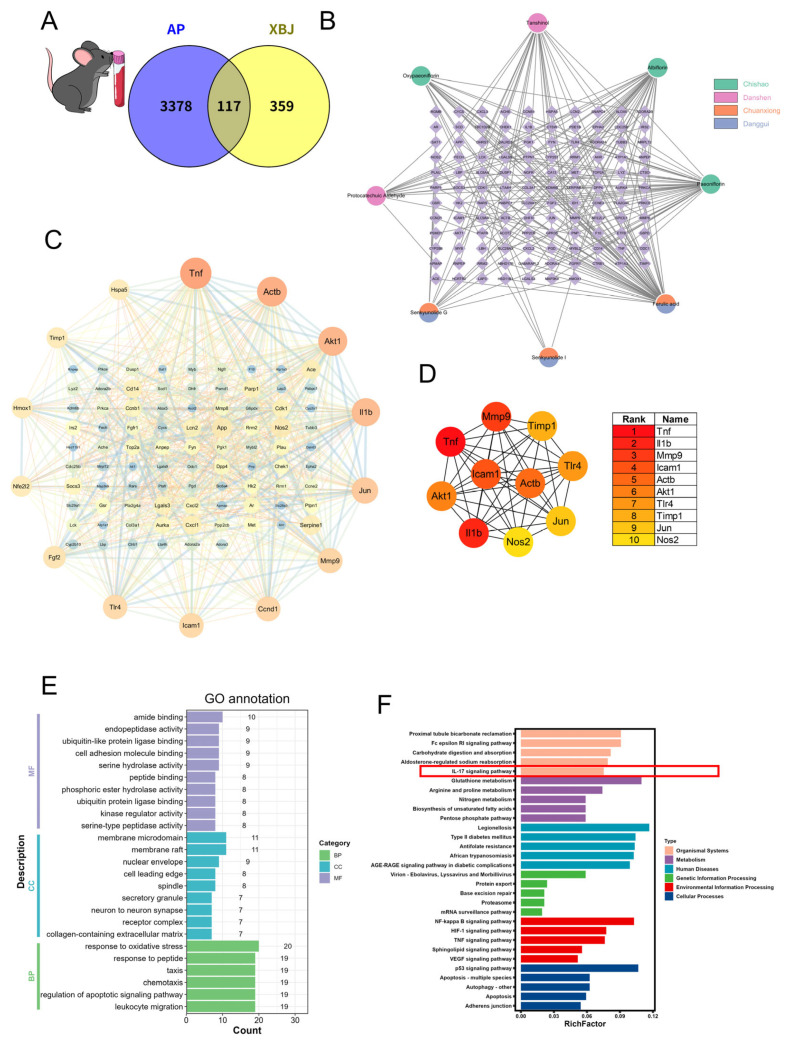
Network pharmacology analysis of XBJ in SAP based on mouse peripheral blood transcriptomics. (**A**) Intersection of “XBJ compound targets” and “SAP targets” in the mouse blood. (**B**) The compound–target network in the mouse blood. (**C**) PPI network. (**D**) Targets of the hub gene in mouse blood during SAP. (**E**) GO functional annotation. (**F**) KEGG pathway enrichment analysis.

To further validate the previous findings, we conducted a detailed analysis of the blood transcriptome from mouse models of SAP to identify DEGs linked to potential XBJ targets. A total of 3495 DEGs associated with SAP were identified, which are primarily involved in immune regulation and inflammatory pathways central to the pathogenesis of SAP (Figure S3A–D). After analysis, we identified 117 common proteins shared between XBJ targets and the DEGs from the SAP model (Figure 6A). The associated compounds and herbs targeting these proteins are displayed in Figure 6B. The PPI network of these 117 proteins was constructed to explore their interactions, with key relationships highlighted in Figure 6C. The CytoHubba plugin was utilized to identify 10 key hub genes, including *Tnf*, *Il1b*, *Mmp9*, *Icam1*, *Actb*, *Akt1*, *Tlr4*, *Timp1*, *Jun*, and *Nos2* (Figure 6D). Further GO and KEGG enrichment analyses demonstrated significant associations with pathways such as “response to oxidative stress”, “leukocyte migration”, “NF-κB signaling pathway”, “TNF signaling pathway” and “IL-17 signaling pathway” (Figure 6E,F), all of which are implicated in the inflammatory processes of SAP.

### 2.7. Network Pharmacology Analysis of XBJ in SAP Based on Mouse Lung Tissue Transcriptomics

To investigate how XBJ alleviates lung injury in SAP, we conducted a comprehensive analysis of the lung transcriptome in the mouse SAP model, aiming to identify DEGs linked to potential XBJ targets. A total of 4910 DEGs were identified, primarily involved in immune regulation and inflammatory pathways that play key roles in the pathogenesis of SAP (Figure S3E–H). Among these, 126 common proteins were found to overlap between XBJ targets and the DEGs from the SAP model (Figure S4A). The compounds and herbs targeting these proteins are illustrated in Figure S4B. PPI network was then generated to examine the interactions between these 126 proteins, with the critical connections depicted in Figure S4C. Using the CytoHubba plugin, we identified 10 hub genes, including *Tnf*, *Il6*, *Il1b*, *Mmp9*, *Tgfb1*, *Tlr4*, *Icam1*, *Akt1*, *Jun*, and *Mapk14* (Figure S4D). Further GO and KEGG enrichment analyses demonstrated significant associations with pathways such as “regulation of inflammatory response”, “leukocyte migration”, “IL-17 signaling pathway”, “TNF signaling pathway”, and “NF-κB signaling pathway” (Figure S4E,F), all of which are closely associated with the pathways involved in the pathogenesis of SAP.

### 2.8. Therapeutic Effects of XBJ in SAP: Hub Targets Recognition, Molecular Docking, and Immune Cell Infiltration

**Figure 7:**
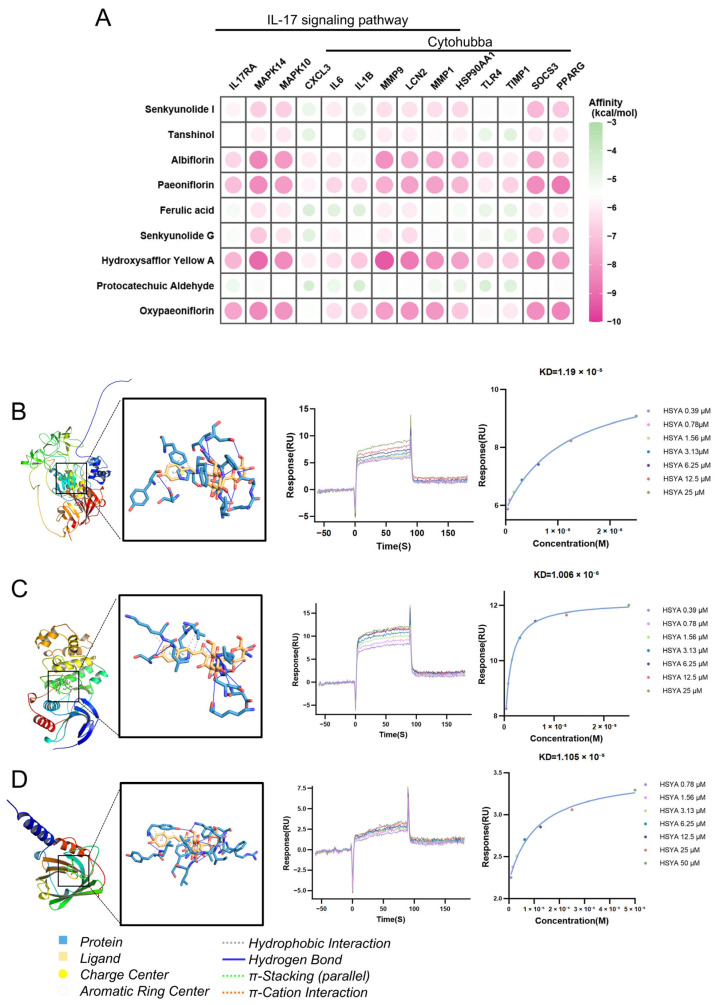
Molecular docking results of targets of SAP with XBJ. (**A**) Molecular docking heatmap of 9 components from XBJ and 14 SAP-related target proteins with the most stable conformation and minimum binding energy. (**B**) The binding conformation of hydroxysafflor yellow A with MMP9. (**C**) The binding conformation of hydroxysafflor yellow A with MAPK14. (**D**) The binding conformation of hydroxysafflor yellow A with LCN2.

Based on the drug-target enrichment analysis of XBJ, as well as the network pharmacology analysis in human blood, mouse blood, and mouse lungs, the IL-17 signaling pathway is suggested to be one of the core mechanisms of XBJ in the treatment of SAP. The proteins associated with the IL-17 signaling pathway in the human blood network, combined with the hub targets identified through CytoHubba, collectively formed 14 core targets (IL17RA, MAPK14, MAPK10, CXCL3, IL6, IL1B, MMP9, LCN2, MMP1, HSP90AA1, TLR4, TIMP1, SOCS3, PPARG) for XBJ in the treatment of SAP. Receiver Operating Characteristic (ROC) curve analysis (Figure S5) revealed strong differentiating potential for SAP from healthy volunteers for most hub genes, with Area Under the Curve (AUC) values greater than 0.75 (e.g., PPARγ: AUC = 0.939, MMP9: AUC = 0.902, MAPK14: AUC = 0.842, SOCS3: AUC = 0.831, IL6: AUC = 0.808, TIMP1: AUC = 0.797, LCN2: AUC = 0.776, IL17RA: AUC = 0.764, IL1B: AUC = 0.758, HSP90AA1: AUC = 0.751), indicating their robust diagnostic or prognostic utility. These findings underscore the potential of XBJ to target these core molecules, providing a novel therapeutic strategy for the effective differentiation and treatment of SAP. These core targets were then subjected to molecular docking analysis with each of the 9 components of XBJ. Molecular docking analysis predicted that the 9 components of XBJ could bind to most of their core targets. Key targets involved in these interactions included IL17RA, MAPK14, MAPK10, MMP9, LCN2, MMP1, HSP90AA1, SOCS3, and PPARG (Figure 7A). The binding energies were further ranked, and the top three docking sites with the lowest binding energies were selected for visualization using PyMOL. The top three interactions with the lowest binding energies are between HSYA and the three potential target proteins: MMP9 (−9.389 kcal/mol; Figure 7B), MAPK14 (−9.046 kcal/mol; Figure 7C), and LCN2 (−8.689 kcal/mol; Figure 7D). Interestingly, although HSYA was not identified as an SAP-related target in the database, it displayed a strong interaction with the potential key targets associated with SAP. Furthermore, SPR analysis verified the direct binding of HSYA to the three putative target proteins. The interactions with MMP9 (KD =1.19 × 10^−5^ M; Figure 7B), MAPK14 (KD = 1.006 × 10^−6^ M; Figure 7C), and LCN2 (KD = 1.105 × 10^−5^ M; Figure 7D) were all characterized by “fast binding/fast dissociation” kinetics, confirming a direct physical interaction.

Based on network pharmacology, XBJ treatment primarily dampens the inflammatory response. Therefore, we further explored the relationship between immune cell infiltration levels in SAP patients and healthy controls using CIBERSORT analysis. Figure S6A illustrates the distribution of various immune cells in each blood sample from the SAP and healthy control groups. Neutrophils were found to represent the largest proportion and their levels increased with SAP. The correlations between hub genes and immune cells are shown in Figure S6B, where significant positive and negative correlations between the hub genes and various immune cell types are largely consistent. Notably, there were significant changes in the core targets, particularly with neutrophils, follicular helper T cells, γδT cells, M0 & M2 macrophages, natural killer cells, memory CD4+ T cells, monocytes, naive B cells, and resting dendritic cells. The correlations between different immune cells are shown in Figure S7A. The differences in hub genes between SAP patients and healthy controls are further illustrated in Figure S7B.

### 2.9. XBJ Treatment Attenuates Severity of SAP Partially via Suppressing IL-17-Related Signaling Pathways

**Figure 8:**
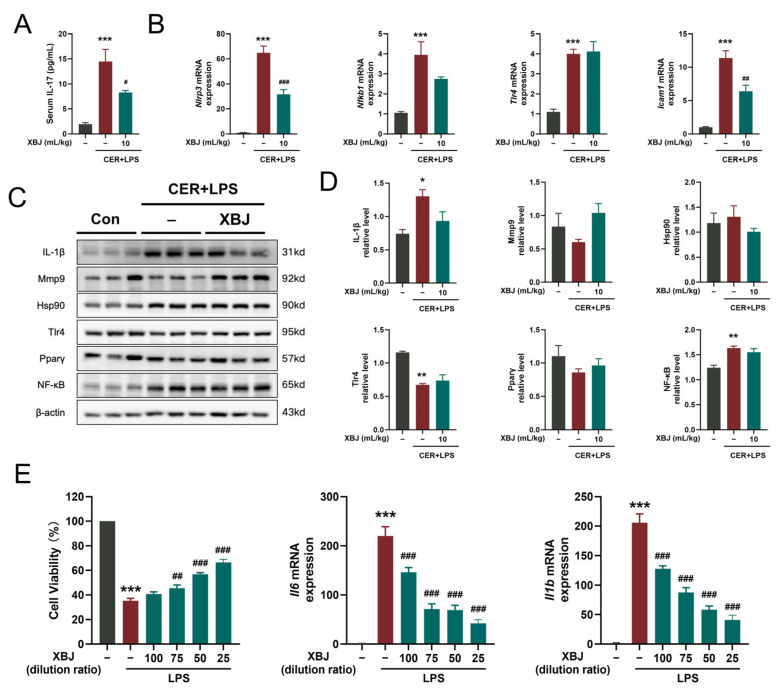
XBJ treatment attenuates severity of SAP mice partially via suppressing IL-17 signaling pathway. (**A**) Levels of serum IL-17 (*n* = 6 per group). (**B**) Expression of proinflammatory marker mRNAs (*Nlrp3*, *Nfκb1*, *Tlr4*, and *Icam1*; *n* = 3–4 per group). (**C**) Representative Western blot images for IL-17 signaling pathway-related proteins and proinflammatory proteins in the pancreatic tissues. (**D**) Quantification of Western blot images for these proteins for the pancreatic tissues (*n* = 4 per group for original immunoblots). (**E**) Protective effect of XBJ against LPS-induced injury in RAW264.7 macrophages. All data are presented as means ± SEM. * *p* < 0.05, ** *p* < 0.01, and *** *p* < 0.001 indicate SAP vs. Control; ^#^ *p* < 0.05, ^##^ *p* < 0.01, and ^###^ *p* < 0.001 indicate SAP + XBJ vs. SAP.

To verify that XBJ treats SAP by acting on the IL-17 signaling pathway, we further examined levels of IL-17 in serum (Figure 8A) and pancreatic tissues (Figure 8B) from SAP mice. The results demonstrate that at the onset of SAP, there was a significant increase in the levels of inflammatory mediators involved in the IL-17 signaling pathway, including serum IL-17 level, and pancreatic *Nlrp3*, *Nfkb*, *Tlr4*, and *Icam1* mRNA levels. Treatment with XBJ was found to significantly reduce the expression of most of these inflammatory factors (*Nlrp3*, *Nfkb*, and *Icam1*). Collectively, our results suggest that XBJ may exert inhibitory effects on IL-17–associated cytokines, including IL-17, IL-1β, TNF-α, and IL-6, at the systemic level, as evidenced by their reduced levels in serum. However, comparable regulatory effects were not observed in pancreatic tissue, as determined by Western blot analysis. Interestingly, XBJ treatment was associated with a downward trend in the expression of several key proteins in the pancreas—namely IL-1β, MMP9, HSP90, TLR4, PPARγ, and NF-κB—all of which are functionally linked to the IL-17 signaling pathway (Figure 8C,D). Despite this trend, the differences did not reach statistical significance, highlighting the need for further investigation to confirm these tissue-specific effects. In an in vitro experiment, we studied RAW264.7 macrophages to investigate the therapeutic mechanisms of XBJ. The results showed that XBJ significantly increased cell viability, exhibiting a concentration-dependent protective effect, especially after LPS-induced inflammatory injury. Additionally, XBJ reduced the secretion of the pro-inflammatory cytokines IL-6 and IL-1β in a concentration-dependent manner (Figure 8E).

### 2.10. Proteomic Analysis of XBJ Treatment in SAP Mice

**Figure 9:**
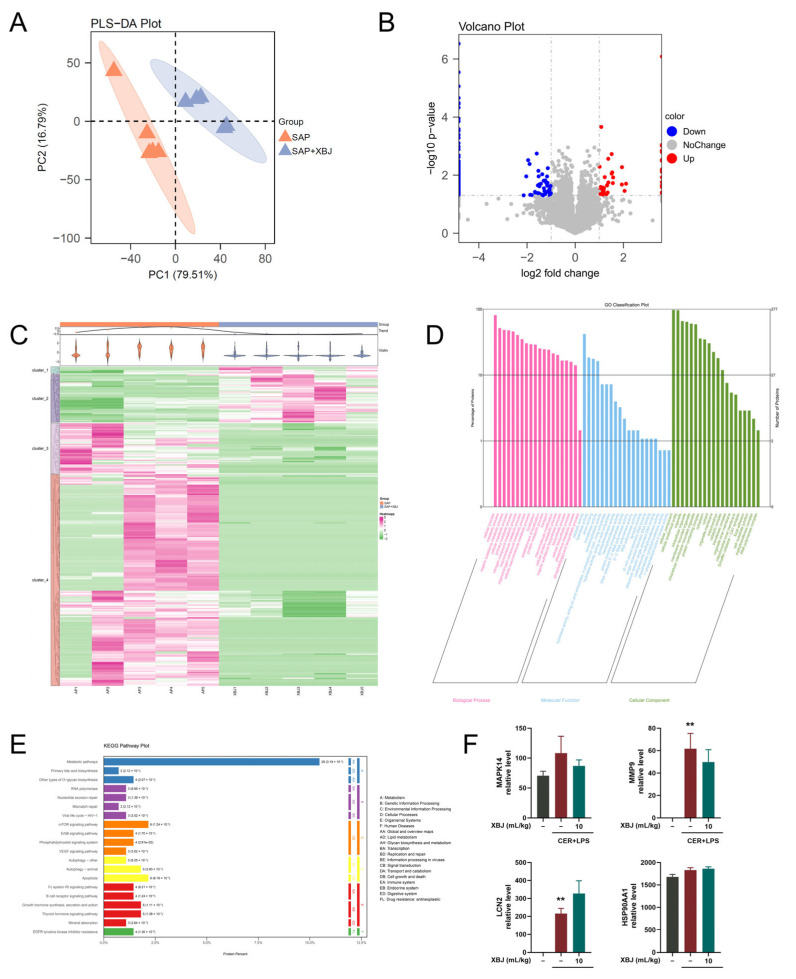
Proteomics screening data of pancreatic tissue from mice protected by XBJ. (**A**) Scores plots for PLS-DA of proteomics profiling data of SAP + XBJ and SAP groups. (**B**) Quantitative volcano plots of DEPs of SAP + XBJ and SAP groups. (**C**) Combined heatmap and trend change plot of DEPs. (**D**) Bar chart of GO enrichment. (**E**) KEGG pathway enrichment. (**F**) Proteomics quantification analysis of detected proteins in hub targets. All data are presented as means ± SEM of 5 samples per each experimental group. ** *p* < 0.01 indicates SAP vs. Control.

To explore the systemic effects of XBJ treatment on SAP, we further conducted a proteomic analysis of pancreatic tissues. We performed partial least squares discriminant analysis (PLS-DA) to evaluate the protein expression profiles from the SAP group and SAP + XBJ group. The multivariate analysis revealed distinct protein expression patterns associated with both SAP induction and XBJ treatment (Figure 9A). In the 10 samples from the two groups, a total of 4595 quantifiable proteins were identified. Among these, 277 DEPs were found between the SAP group and the XBJ treatment group. Specifically, 48 proteins were upregulated in the XBJ treatment group, while 229 proteins were downregulated compared to the SAP group (Figure 9B). The combined heatmap and trend change plot reveal significant differential expression of 277 proteins, exhibiting distinct expression patterns and trends when compared to the SAP group (Figure 9C). To further understand the functional significance of these altered proteins, we performed pathway enrichment analysis using bioinformatics tools such as GO and KEGG (Figure 9D,E). The results indicated that XBJ treatment significantly impacted several important pathways in the context of SAP, such as apoptosis, autophagy, mTOR signaling, and others. In accord with the immunoblot results, most of our hub targets were not detected in pancreatic tissue proteomics; and yet among those proteins detected there were no statistically significant differences in proteomics quantification (Figure 9F).

These findings enabled us to define a proteomics signature that reflects the biological actions of XBJ, revealing the molecular changes induced by XBJ treatment. Through mapping these alterations and together with the transcriptomics and network pharmacology analyses, we gained a clearer understanding of the potential therapeutic mechanisms of XBJ in SAP.

## 3. Discussion

In this study, we evaluated the efficacy of the injectable CHM formula—XBJ in treatment of experimental SAP. We further detailed the principal active components of XBJ (HSYA, paeoniflorin, albiflorin, oxypaeoniflorin, senkyunolide I, senkyunolide G, ferulic acid, tanshinol, and protocatechuic aldehyde), and documented the protective effect of the XBJ against pancreatic toxins in vitro. We employed an innovative approach that combined proteomics and multi-species transcriptomics (data sourced from human and mouse blood, as well as mouse lung) with network pharmacology to explore the mechanisms of action of XBJ in the treatment of SAP. This strategy identified 14 key targets, the most prominent of which was IL-17-related signaling. Molecular docking predicted high binding affinities between all 9 active compounds of XBJ and potential key targets, with HSYA exhibiting the strongest interactions. Proteomics and immunoblots indicated that XBJ affects SAP development through apoptosis, autophagy, and mTOR signaling, rather than directly modulating IL-17 signaling within pancreatic tissue. Thus, XBJ’s therapeutic effects in SAP are primarily through suppressing systemic IL-17-related inflammation.

XBJ is currently widely used in China for a variety of inflammatory diseases, including sepsis, pneumonia, ischemic stroke, and even COVID-19 [8]. These diseases, along with SAP, share significant pathophysiological similarities, such as the amplification of local cell death leading to systemic inflammation and multiple organ failure [9,10]. SAP is a classical model for studying this cell death to organ failure feed-forward loop, where injured PACs release cytokines (TNF-α, IL-1β, and IL-6), chemokines, and damage associated molecular pattern molecules locally or into circulation, to promote neutrophil and monocyte infiltration [11]. Our study shows that XBJ protects PACs from pancreatitis toxin, potentially reducing subsequent pro-inflammatory signals and disease severity. XBJ also mitigates pancreatic and lung injury, as evidenced by histopathology score, inflammatory mediators, and tissue myeloperoxidase levels. These findings correlate with results from animal studies in multiple other diseases including acute liver injury [12], sepsis [13], and COVID-19-induced cardiac dysfunction [14].

With the advancement of computational technologies and systems biology, network pharmacology has emerged as a powerful tool to investigate molecular mechanisms involving multiple complex interactions such as with CHM [15], helping identify novel therapeutic pathways and targets. This transcriptomics and proteomics approach complemented by molecular docking studies, enabled us to identify several key hub targets potentially attributed to the therapeutic action of XBJ in SAP. Of these targets, the roles of IL17RA, IL6, IL1B, TLR4, SOCS3, and PPARG have been well described in exacerbating severity of AP [16,17,18]. Our study also identified other contributors to disease severity with perhaps a less established evidence base, including MAPK14, MAPK10, CXCL3, MMP9, LCN2, MMP1, HSP90AA1, and TIMP1. Critically, all these targets influence the IL-17-related signaling pathways, making this a prominent target for XBJ.

IL-17 signaling is a key orchestrator within the immune network, linking adaptive and innate immunity in various inflammatory disorders, including conditions for which XBJ is a therapy [19]. Produced primarily by T helper 17 cells [20], IL-17 activates various downstream signaling pathways like NF-κB, MAPK and AKT that lead to the production of pro-inflammatory cytokines such as TNF-α, IL-1β, and IL-6 [19,21]. This cascade drives neutrophil recruitment and contributes significantly to the pathogenesis of inflammatory diseases [22]. Previous studies have reported that IL-17 can exacerbate inflammation in the lung caused by sterile injury, pneumonia, sepsis, influenza virus, and COVID-19 [19]. Given that the lung is the commonest organ to fail in SAP [3], and IL-17 exacerbates pulmonary inflammation in similar conditions, its modulation is critical. Our study demonstrates that XBJ reduced lung histopathology, pro-inflammatory markers, and myeloperoxidase activity in SAP mice by modulating IL-17, TNF-α, and NF-κB pathways. This protective effect aligns with similar mechanistic findings recently reported for XBJ in models of pneumonia and sepsis [23].

Proteomic analysis of pancreatic tissue demonstrated that XBJ treatment modulated several key pathways implicated in SAP pathogenesis, including apoptosis, autophagy, and mTOR signaling [24]. These pathways are closely linked to IL-17 signaling, which orchestrates inflammation and contributes to SAP progression [25,26,27,28]. Specifically, XBJ downregulated key pro-inflammatory cytokines (TNF-α, IL-1β, and IL-6) and inhibited crucial inflammatory pathways (IL-17 and NF-κB). Therefore, XBJ not only suppresses circulatory and lung inflammatory responses, but it also modulates the pancreatic immune milieu. It is likely the combination of these effects that leads to the observed reduction in severity of SAP in XBJ-treated mice. However, several IL-17-related signaling proteins did not significantly differ in immunoblots or proteomics between the SAP + XBJ and SAP untreated groups. We propose that the inhibitory effect of XBJ on IL-17-related signaling pathways may primarily originate from systemic immune compartments—such as circulating or infiltrating immune cells—rather than pancreatic parenchymal cells. This hypothesis aligns with IL-17’s role as a systemic orchestrator of inflammation and is supported by the lack of significant modulation within pancreatic tissue, which may be attributable to pharmacokinetic limitations like the blood–pancreas barrier. Therefore, XBJ likely exerts its therapeutic effects through distinct yet complementary mechanisms in the circulation, lung, and pancreas.

This study has several limitations. Firstly, the identification of active compounds relied on broad screening and quality control of known bioactive agents, a broad approach that may have overlooked compounds specifically effective in SAP. Secondly, virtual prediction of potential drug targets is subject to limitations. The observed discrepancy—where HSYA showed high binding affinity in molecular docking despite having few targets predicted by public databases—suggests that in silico tools may not fully capture the therapeutic potential of all active compounds. Thirdly, the current scope lacks immediate functional validation; while HSYA binding was confirmed by physical assays (molecular docking and SPR), the downstream functional assays necessary to confirm the biological impact of this binding were not performed, which are the explicit focus of our planned follow-up investigation. Fourthly, the optimal XBJ dose (10 mL/kg) was selected primarily based on histopathological scoring, which confirmed its in vivo efficacy. However, a more comprehensive dose–response assessment utilizing molecular markers, such as IL-17 pathway proteins, is necessary to fully elucidate the deeper mechanistic basis of XBJ’s therapeutic effects. Further investigation is needed to precisely delineate the functional mechanisms attributable to each active component of XBJ.

## 4. Materials and Methods

### 4.1. Ethics and Animals

Adult male C57BL/6J mice, aged 8–10 weeks and weighing 25 ± 1 g, were purchased from Beijing Huafukang Bioscience Co., Ltd. (Beijing, China). The mice were housed under controlled conditions, with a temperature of 22 ± 2 °C and a 12-h light/dark cycle. They were provided with standard laboratory chow and had unrestricted access to food and water before and throughout the experiments. The study protocol was thoroughly reviewed and approved by the Animal Ethics Committee of West China Hospital, Sichuan University (No. 20230412003). All experimental procedures were conducted in strict accordance with the relevant ethical guidelines and regulations governing animal research.

### 4.2. Materials and Reagents

XBJ was purchased from Tianjin Hongri Pharmaceutical Co. (Tianjin, China; Batch No. 2406232). Standards for hydroxysafflor yellow A, paeoniflorin, albiflorin, oxypaeoniflorin, senkyunolide I, senkyunolide G, ferulic acid, tanshinol, and protocatechuic aldehyde, all with a purity of ≥98%, were obtained from Chengdu Push Bio-technology Co., Ltd. (Chengdu, China). Commercial ELISA kits for serum IL-17, TNF-α, IL-1β, and IL-6 were from R&D Systems, Inc. (Shanghai, China). The antibodies were from CST (Shanghai, China), anti-β-actin antibody was from Proteintech (Wuhan, China). For the surface plasmon resonance experiment, hydroxysafflor yellow A and the recombinant protein MMP9/MAPK14/LCN2 were purchased from TargetMol Chemicals (Shanghai, China). If not otherwise stated, all other reagents were purchased from Sigma-Aldrich (Shanghai, China).

### 4.3. UPLC-QTOF/MS Analysis of XBJ and Standards

We followed the novel assays for quality evaluation of XBJ developed previously [29] and conducted UPLC-QTOF/MS analysis to identify the compounds present in XBJ. The XBJ stock solutions (5-, 10-, and 100-fold dilution) were prepared. The above nine standards were dissolved in dimethyl sulfoxide and then mixed in a certain proportion for detection. A UPLC single bondClass with a Waters SYNAPT G2-Si HDMS Q-TOF (Waters Corporation; Milford, MA, USA) was used to analyze the XBJ solution and standards under optimized chromatography conditions. All ingredients were separated in 8 min on a BEH C18 column (2.1 × 100 mm, 1.7 μm) at 35 °C. The elution gradient was 0.1% formic acid solution (A) and methanol (B) at a flow rate of 0.3 mL/min as follows: 0–3.0 min, 5% B; 3.0–5.5 min, 5–40% B; 5.5–6.5 min, 40–80% B; 6.5–6.6 min, 80–5% B; 6.6–8 min, 5% B. The injection volume was 1 μL. The Q-TOF system operated in positive and negative ion modes with an electrospray ion source. The mass range was *m*/*z* 50–1200. The capillary voltage was 1.0 KV. The source temperature was 120 °C and the desolvation temperature was 400 °C. Cone gas flow and desolvation gas flow were 50 and 800 L/h, respectively. Data was acquired in MSE mode and analyzed using UNIFI 1.9.2 software (Waters Corporation).

### 4.4. SAP Model Induction and Treatment

Mice were randomly assigned into five groups. The sample size (*n* = 6 per group) was determined based on data variability and expected effect sizes observed in similar studies published in the literature [30]. Mice received a single intraperitoneal injection of cerulein (50 μg/kg) per hour seven times, immediately followed by a single injection of lipopolysaccharide (LPS, 10 mg/kg) to establish an experimental SAP model mimicking necrotizing pancreas with septic shock. In the treatment groups, two intraperitoneal injections of different doses of XBJ (2.5, 5, and 10 mL/kg) were conducted, starting from the third cerulein injection and repeated 5 h later. Control mice received normal saline injections at the same regimen of SAP group. Mice in all experimental groups were sacrificed at 12 h after the first cerulein or saline injection. For humane euthanasia, mice were placed into an induction chamber and euthanized by continuous exposure to an overdose concentration of the volatile anesthetic, isoflurane. Blood and tissue samples were immediately collected for processing and storage before subsequent analyses. All animals that survived until the study endpoint were included in the final analysis.

### 4.5. Histological Analysis and Immunostaining

Pancreas and lung tissues were collected, fixed, and stained with H&E for histological examination. Histopathological scoring was subsequently carried out by two independent, blinded observers. Immunohistochemistry was performed for lung myeloperoxidase as we previously reported [31].

### 4.6. Serum Cytokine Profiling

The levels of serum IL-17, TNF-α, IL-1β, and IL-6 were determined by respective ELISA kit following manual instructions.

### 4.7. RT-qPCR and Western Blotting

Detailed protocols are available in our previous studies [31]. Briefly, total RNA from pancreatic and lung tissues was extracted using TRIzol and reverse-transcribed with M-MLV (Thermo Fisher, Waltham, MA, USA). RT-qPCR was performed with SYBR Green (Vazyme, Nanjing, China), and expression levels were normalized to 18S rRNA using the 2^ΔΔCt^ method. Proteins were extracted with RIPA buffer, quantified by Bradford assay, and analyzed by Western blot using antibodies against IL-1β (CST:12242S, 1:1000), TLR4 (Santa:sc-293072, 1:500), MMP-9 (CST:13667, 1:1000), HSP90 (CST:4877, 1:1000), PPARγ (CST:2435, 1:1000), NF-κB (CST:8242,1:1000) and β-actin(Proteintech:66009-1-lg, 1:10,000).

### 4.8. Necrotic Cell Death Assay

Pancreatic acinar cells (PACs) were freshly isolated from mice following established protocols we previously reported. The isolated PACs were then pre-treated with varying dilutions of XBJ for 30 min. Immediately following pre-treatment, the cells were incubated with commonly used pancreas toxins [32]—taurolithocholic acid 3-sulfate disodium salt (TLCS; 500 μM) or cerulein (500 nM) for an additional 30 min. After the treatment, the PACs were washed to remove excess reagents. The working concentrations of XBJ (100-, 75-, 50-, and 25-fold dilutions) were selected based on previously reported in vitro studies and were applied to explore potential dose–response relationships in PACs. Necrotic cell death was quantified by calculating the percentage of PI (propidium iodide)-positive cells relative to DAPI (4′,6-diamidino-2-phenylindole)-stained cells.

Murine RAW264.7 macrophages were pre-incubated with XBJ (100-, 75-, 50-, and 25-fold dilutions) for 2 h, subsequently stimulated with LPS (1 μg/mL) for 24 h, and cell viability was quantified via CCK-8 assay.

### 4.9. Transcriptome Analysis of Peripheral Blood from SAP Patients

The high-throughput bulk sequencing data associated with acute pancreatitis patients came from the GSE194331 dataset in the Gene Expression Omnibus (GEO, http://www.ncbi.nlm.nih.gov/geo, accessed on 17 February 2024). The dataset included peripheral blood gene expression data from healthy controls (*n* = 32) as well as patients with acute pancreatitis (*n* = 87). Dependent on presence or absence of local complications and/or persistent organ failure defined by the Revised Atlanta Classification [33], they were further classified into SAP (*n* = 30) and non-SAP (*n* = 57), respectively. Raw gene counts were converted from pooled gene names to gene symbols using the org.Hs.eg.db package in R. Analysis of differentially expressed genes (DEGs) was performed using the DESeq2 package (v1.42.0), with criteria of fold change (|FC|) > 2 and adjusted *p*-value < 0.05. The DEGs were then visualized using R packages pheatmap (v1.0.12), dplyr (v1.1.4), ggplot2 (v4.0.0) to plot heatmaps and volcano plots.

### 4.10. Transcriptome Analysis of Blood and Lung Tissue from SAP Mice

The raw RNA-seq data from SAP mice (cerulein and LPS regimen as we reported) are available in the GEO database (GSE244335) and include data from mouse blood and lung tissues. The analysis pipeline involved filtering raw data and removing adapter sequences with Trim Galore (v0.6.6), quality control with fastp (v0.20.1), alignment to the GRCm38 mouse genome using STAR (v2.7.4a), quantifying gene expression with featureCounts (v2.0.1), and performing differential expression analysis using DESeq2 with threshold of adjusted *p*-value < 0.05 and |FC| > 2.

### 4.11. Functional and Pathway Enrichment Analysis

Enrichment analyses were performed by referring to the Gene Ontology (GO) and Kyoto Encyclopedia of Genes and Genomes (KEGG) databases by using the clusterProfler package (v4.10.1). An adjusted *p*-value of < 0.05 was considered statistically significant.

### 4.12. Network Pharmacology Analysis

For each of the compounds, we based on multiple databases to predict the potential targets: Traditional Chinese Medicine Systems Pharmacology Database and Analysis Platform (TCMSP) [34], The Encyclopedia of Traditional Chinese Medicine (ETCM) [35], HERB database (a high-throughput experiment- and reference-guided database of traditional Chinese medicine) [36], Search Tool For Interactions of Chemicals (STITCH) [37], TM-MC (an enhanced chemical database of medicinal materials in Northeast Asian traditional medicine) [38] and the SwissTargetPrediction databases [39]. No threshold criteria were applied in any of the databases, and all predictions were retrieved using the default parameters provided by each platform. The obtained targets were then merged and deweighted to obtain the final potential targets of XBJ.

The targets of XBJ and DEGs of SAP were plotted by Venn diagram, and the intersection genes were identified as SAP targets regulated by XBJ. Using Cytoscape (v3.7.2) to establish a “compound–target” network. The protein–protein interaction (PPI) network was constructed using the STRING online tool to detect the interaction between intersection genes [40]. Hub genes were further investigated and visualized using the CytoHubba. Significant gene modules were identified using the Molecular Complex Detection (MCODE).

### 4.13. Proteomics of Pancreatic Tissue in SAP Mice

Pancreata were immediately removed after blood withdrawn from the SAP mice. Protein extraction, digestion, and peptide cleanup were performed using the iST Sample Preparation Kit (PreOmics, Planegg, Germany). Following lyophilization, peptides were reconstituted in 0.1% formic acid (FA) and analyzed via an UltiMate 3000 LC system (Thermo Fisher Scientific, Waltham, MA, USA) connected to a timsTOF Pro 2 MS (Bruker Daltonics, Bremen, Germany). A 200 ng sample aliquot was separated on an AUR3-15075C18 column (15 cm length, 75 µm i.d, 1.7 µm particle size, 120 Å pore size, IonOpticks, Collingwood, VIC, Australia) using a 30 min gradient at a flow rate of 400 nL/min. The gradient utilized Buffer B (80% Acetonitrile with 0.1% FA) and ramped from 4% B to 28% in 15 min, 44% in 4 min, 90% in 4 min, held for 3 min, and then equilibrated at 4% for 4 min. Data acquisition was performed in diaPASEF mode, with the scanning range set from *m*/*z* 349 to 1229, and the collision energy was linearly ramped from 59 eV (1/K0 = 1.6 Vs/cm^2^) to 20 eV (1/K0 = 0.6 Vs/cm^2^). Tandem mass spectra were processed using Spectronaut 18 (Biognosys AG, Schlieren, Switzerland) with the UniProt Mus musculus database (version 2022, 21,992 entries), specifying Trypsin digestion and defining fixed Carbamidomethylation and variable Oxidation (M) and Acetylation (Protein N-term) modifications. Protein group quantification was achieved using the MaxLFQ method with a strict FDR cutoff of 1% at both the precursor and protein levels, employing dynamic iRT calibration and local normalization. The differential analysis focused on examining the overall variations in protein expression, identifying differentially expressed proteins (DEPs), and performing clustering analysis on their expression patterns. Proteins with |FC| > 1 and adjusted *p*-value < 0.05 are considered differentially expressed.

### 4.14. Immune Cell Infiltration Analysis in SAP Patients

CIBERSORT (https://cibersortx.stanford.edu/, accessed on 2 March 2024) was applied to evaluate the infiltration status of 22 distinct immune cells between SAP patients and healthy controls. Gene expression matrix data were inputted to CIBERSORT, which included the absolute level of immune infiltration in each sample. Immune cells infiltrate matrix formation. A barplot was plotted using the ggplot2 package to determine immune cell infiltration in each sample. Correlation heatmaps were used to visualize the correlation between hub genes and 22 immune cell infiltration.

### 4.15. Molecular Docking Analysis and Surface Plasmon Resonance (SPR) Assay

Protein structures were queried from the AlphaFold Protein Structure Database, and PDB files were downloaded. These files were processed using AutoDockTools (v1.5.7) for hydrogenation, charge calculation, and atom type addition, then saved in PDBQT format as receptors. Compound SDF files from PubChem were converted to mol2 format using Openbabel (v2.4.1) and further processed in AutoDockTools to PDBQT format as ligands. Molecular docking was performed using AutoDock Vina (v1.1.2) with a blind docking strategy. Docking results were evaluated based on binding affinity scores (kcal/mol), and the top-ranked binding pose was selected for further analysis. Key interactions were visualized using Protein–Ligand Interaction Profiler (PLIP) website [41] and PyMOL (v3.8).

Binding assays were conducted to assess the interaction between the core proteins and hydroxysafflor yellow A (HSYA) using a Biacore X100 instrument (Cytiva, Uppsala, Sweden) at 25 °C with PBS as the running buffer. The protein was first dissolved in sterile double-distilled water and immobilized onto a CM5 chip under specific conditions. For the binding analysis, serially diluted HSYA, starting from a stock concentration of 50 μM, was injected at a flow rate of 10 μL/min for a 90 s contact phase, followed by a 90 s dissociation phase. The binding data were then collected and analyzed using the Biacore X100 evaluation software (v2.0.2).

### 4.16. Statistical Analysis

Data analysis and statistical analysis were performed by R software (v 4.1.3) and GraphPad Prism (v 8.0). Two-tailed Student’s *t*-test was applied to analyze the differences for two-group comparisons. All adjusted *p*-value (adj P) for the omics data were calculated using the Benjamini–Hochberg (BH) procedure to rigorously control the False Discovery Rate (FDR). The *p*-value < 0.05 was considered statistically significant.

## 5. Conclusions

Transcriptomics, proteomics and network pharmacology approaches reveal that XBJ, a drug product of CHM, exerts therapeutic effects on SAP characterized by its multi-component, multi-target, and multi-pathway properties. Experimental validation confirms XBJ effectively reduces pancreatic, lung, and circulatory pro-inflammatory parameters, mainly through inhibiting systemic, but not pancreatic parenchyma, IL-17-related signaling pathways. Our study provides new insights into the treatment of SAP and the therapeutic mechanisms of XBJ, while also suggesting feasible strategies for the research of CHM formulas.

## Data Availability

The original contributions presented in the study are included in the article/Supplementary Material, further inquiries can be directed to the corresponding author. The transcriptome data used in the study are publicly available in the Gene Expression Omnibus (GEO) database under accession numbers GSE194331 and GSE244335. The mass spectrometry proteomics data have been deposited to the ProteomeXchange Consortium (https://proteomecentral.proteomexchange.org, accessed on 14 November 2025) via the iProX partner repository with the dataset identifier PXD070721.

## Supplementary Materials

The following supporting information can be downloaded at: https://www.mdpi.com/article/10.3390/ph18121866/s1, Figure S1. Target prediction of XBJ; Figure S2. Analysis of blood transcriptomics in SAP Patients; Figure S3. Analysis of blood transcriptomics in SAP mice; Figure S4. Network pharmacology analysis of XBJ in SAP based on mouse lung tissue transcriptomics; Figure S5. Hub Targets in Predicting Acute Pancreatitis Risk: Receiver Operating Characteristic (ROC) Curve Analysis; Figure S6. Immune Cell Infiltration in SAP patients; Figure S7. Immune Cell Infiltration in SAP patients.

## Abbreviations

The following abbreviations are used in this manuscript:

CHM	Chinese herbal medicine
DAPI	4′6-diamidino-2-phenylindole
DEGs	differentially expressed genes
DEPs	differentially expressed proteins
ETCM	The Encyclopedia of Traditional Chinese Medicine
FC	fold change
GEO	Gene Expression Omnibus
GO	Gene Ontology
KEGG	Kyoto Encyclopedia of Genes and Genomes
HSYA	hydroxysafflor yellow A
MCODE	Molecular Complex Detection
PACs	Pancreatic acinar cells
PI	propidium iodide
PLS-DA	partial least squares discriminant analysis
PPI	protein–protein interaction
RCTs	randomized controlled trials
SAP	severe acute pancreatitis
STITCH	Search Tool For Interactions of Chemicals
TCMSP	Traditional Chinese Medicine Systems Pharmacology Database and Analysis Platform
TLCS	taurolithocholic acid 3-sulfate disodium salt
TNF-α	tumor necrosis factor-alpha
XBJ	Xuebijing Injection
