# Supplementary figures and images for: Integrated Transcriptomic, Proteomic, and Network Pharmacology Analyses Unravel Key Therapeutic Mechanisms of Xuebijing Injection for Severe Acute Pancreatitis
Source: Pharmaceuticals (Basel). 2025 Dec 7;18(12):1866. doi: 10.3390/ph18121866 (PMC12736240; doi:10.3390/ph18121866)

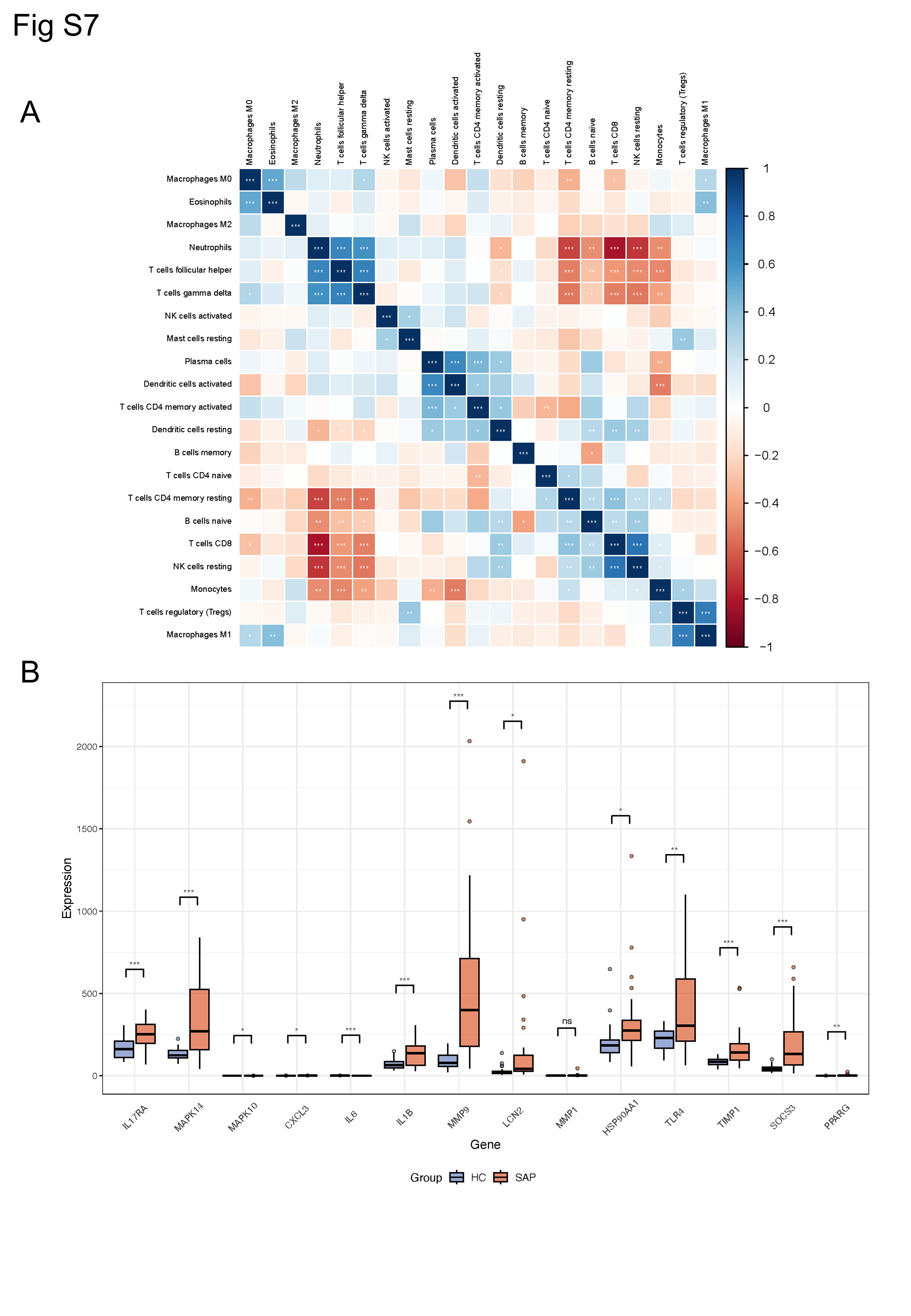

Supplement: Supplementary file 1 [file pharmaceuticals-18-01866-s001.zip › Supplementary Figure/FigureS7.TIF]

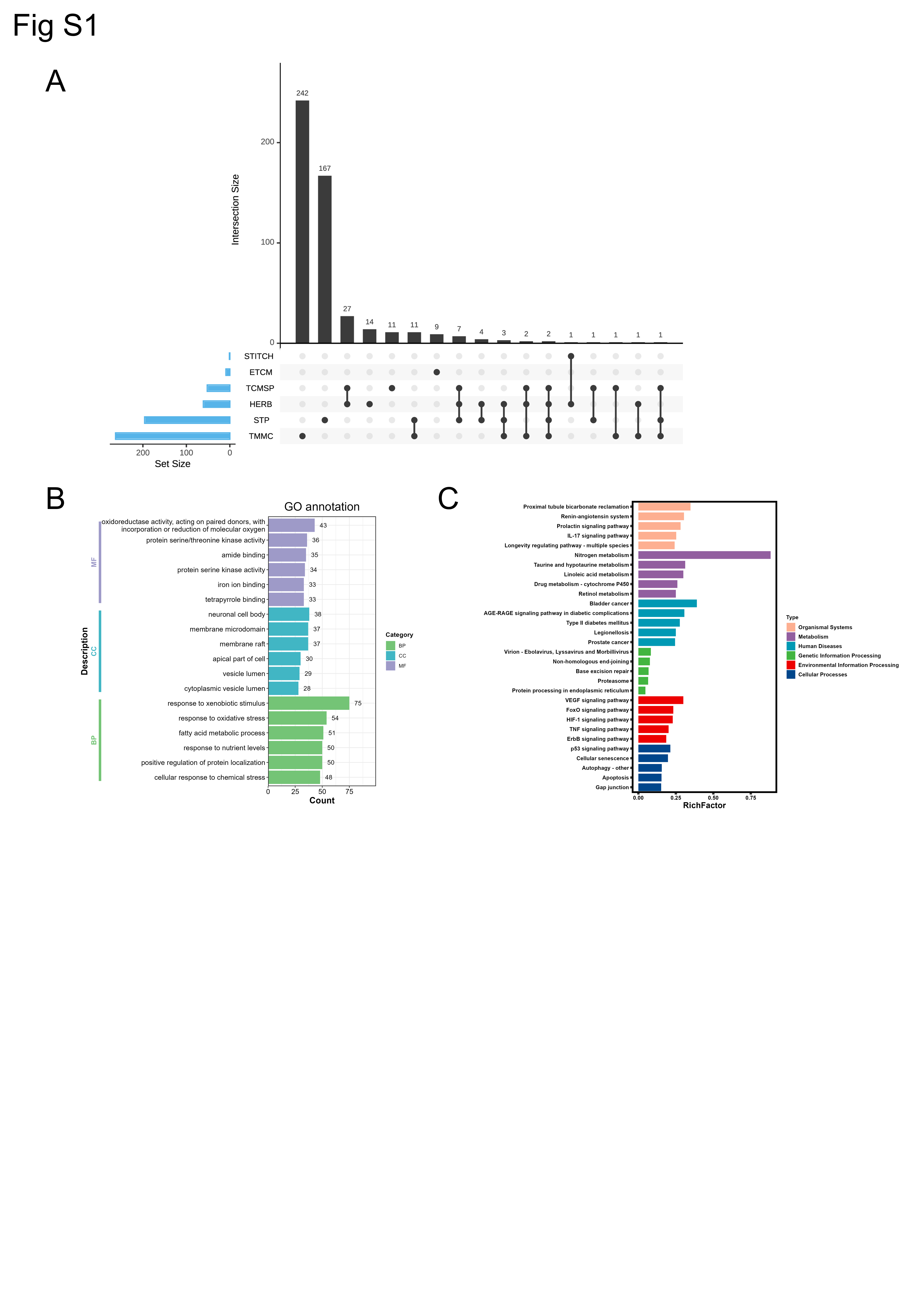

Supplement: Supplementary file 1 [file pharmaceuticals-18-01866-s001.zip › Supplementary Figure/FigureS1.TIF]

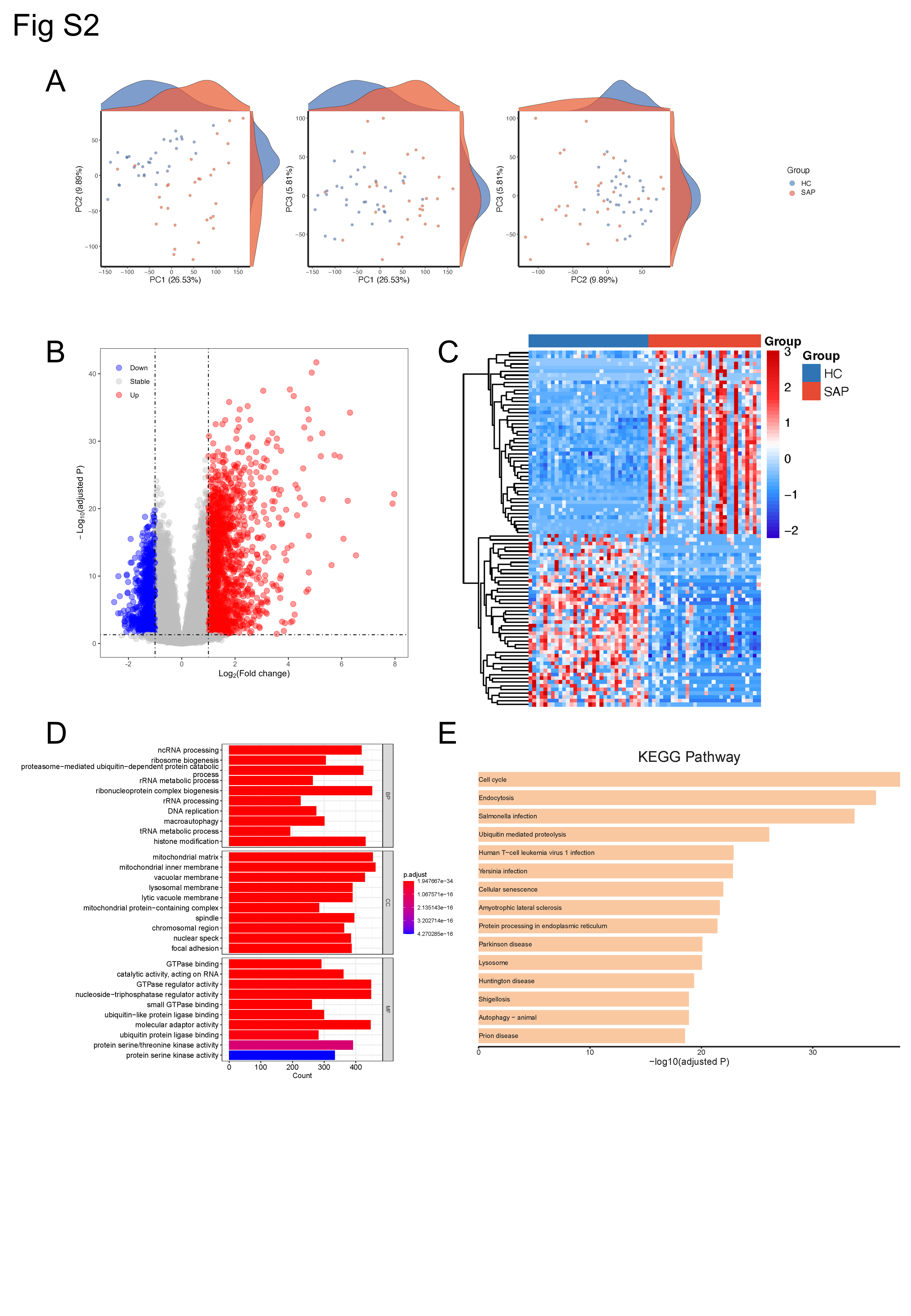

Supplement: Supplementary file 1 [file pharmaceuticals-18-01866-s001.zip › Supplementary Figure/FigureS2.TIF]

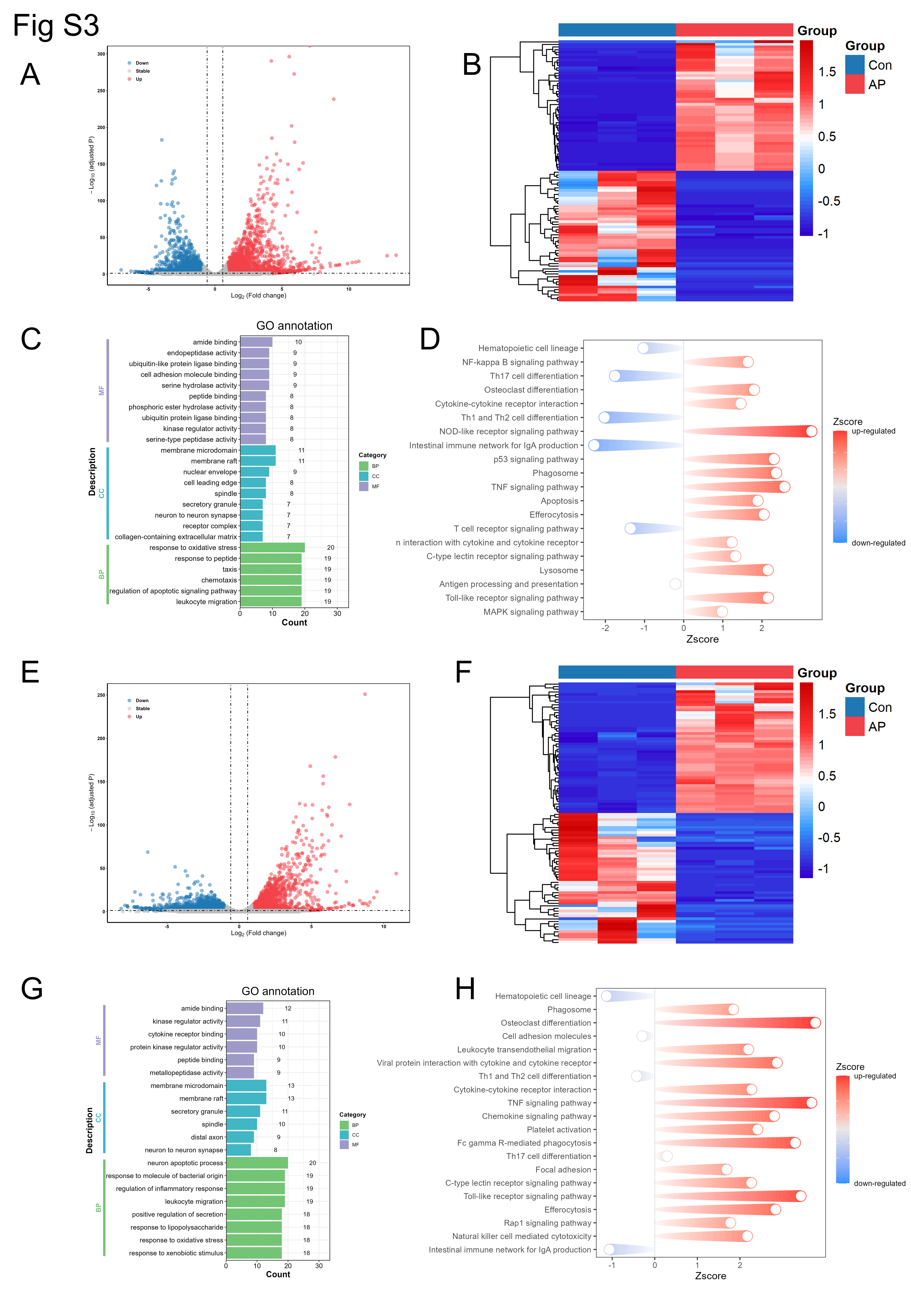

Supplement: Supplementary file 1 [file pharmaceuticals-18-01866-s001.zip › Supplementary Figure/FigureS3.TIF]

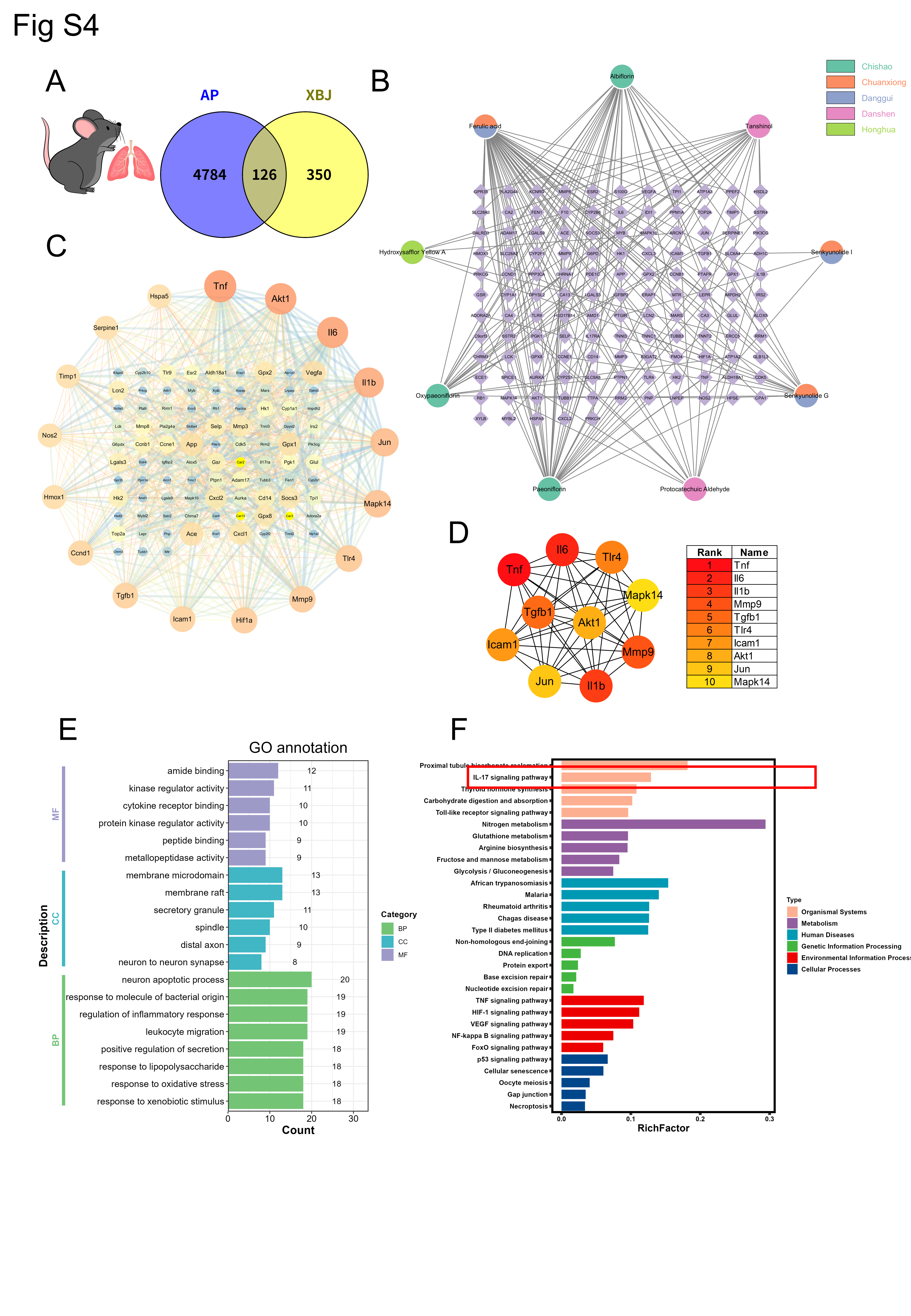

Supplement: Supplementary file 1 [file pharmaceuticals-18-01866-s001.zip › Supplementary Figure/FigureS4.TIF]

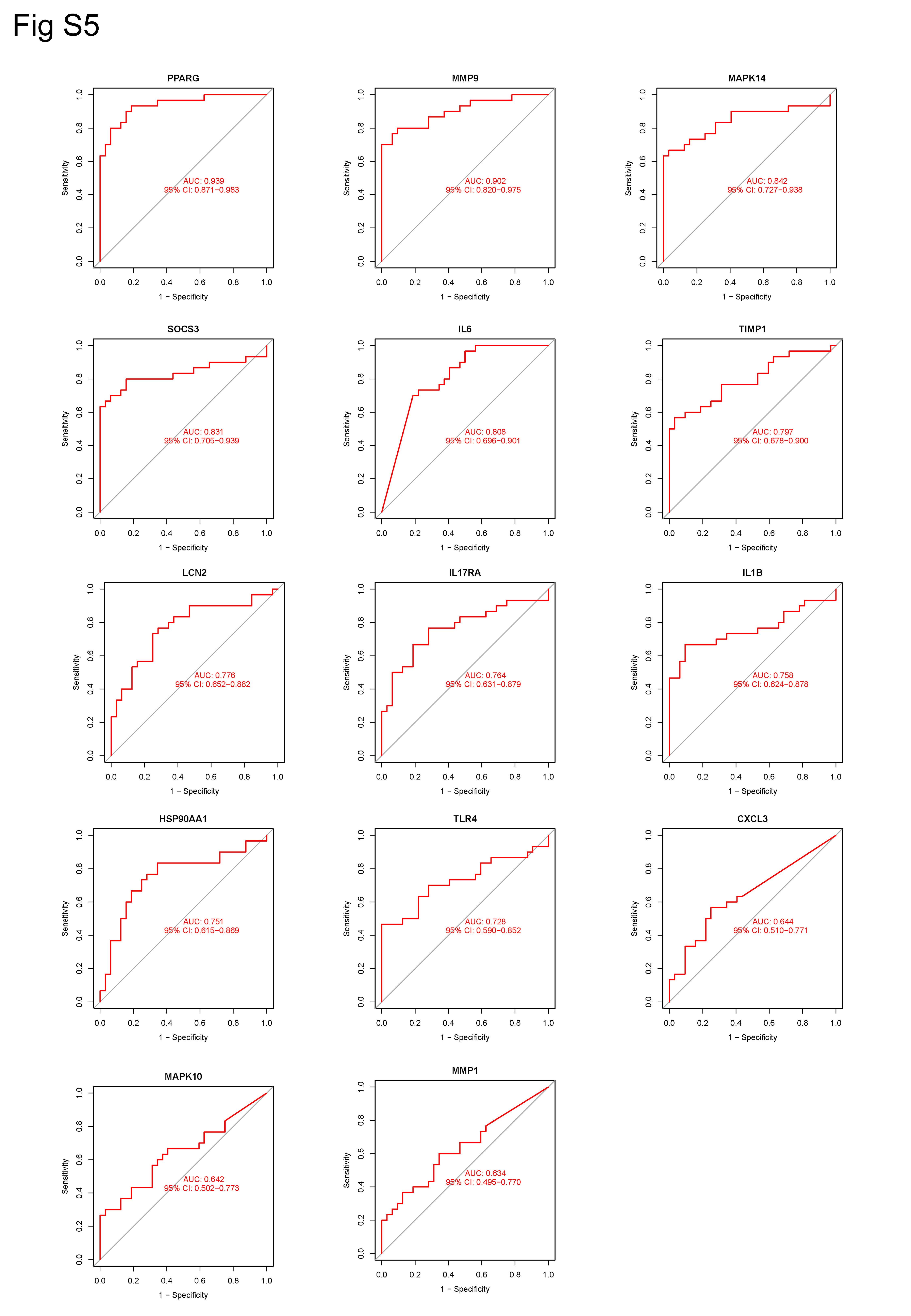

Supplement: Supplementary file 1 [file pharmaceuticals-18-01866-s001.zip › Supplementary Figure/FigureS5.TIF]

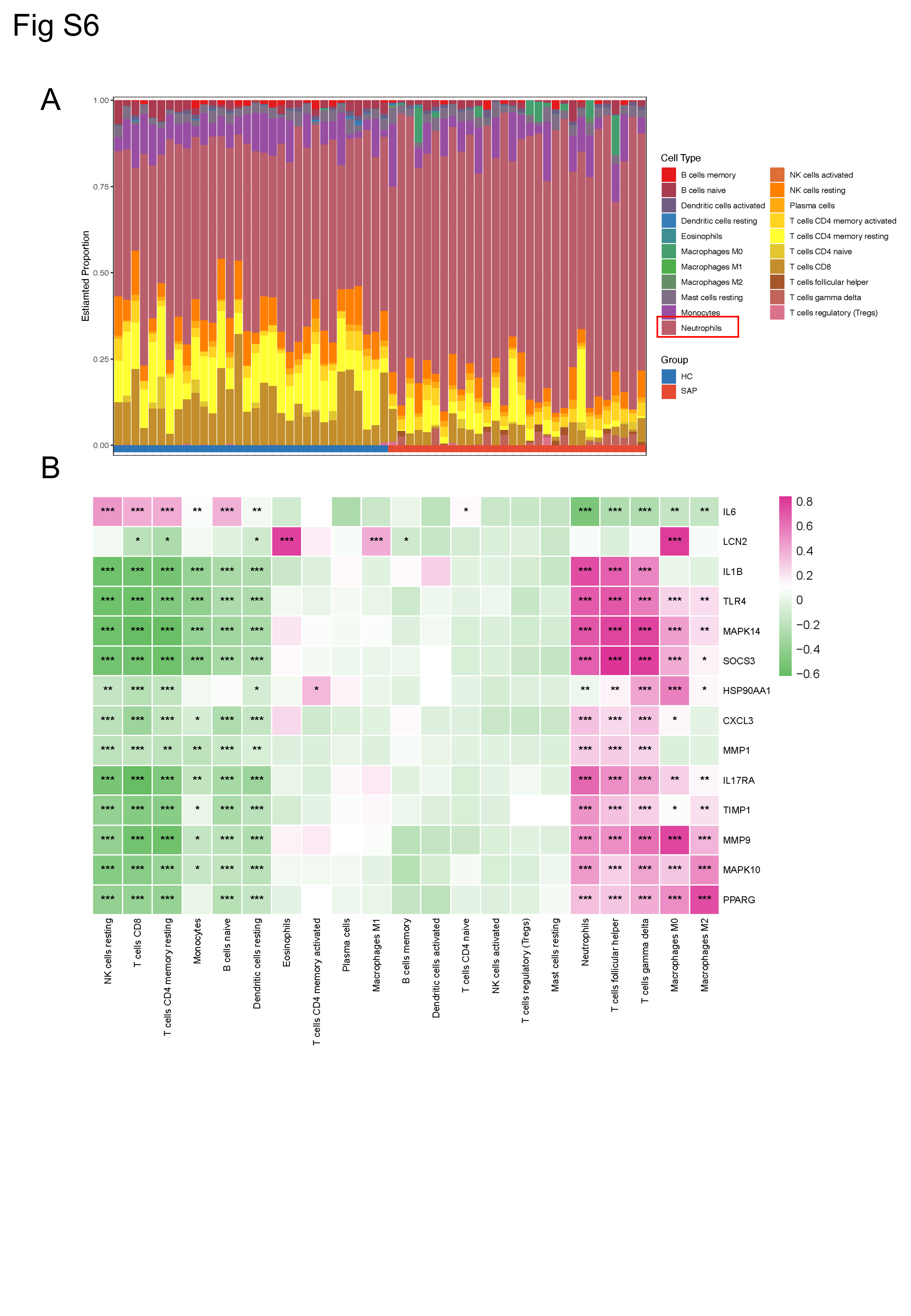

Supplement: Supplementary file 1 [file pharmaceuticals-18-01866-s001.zip › Supplementary Figure/FigureS6.TIF]
